# 985 Social Workers as Members of Burn Care Teams: Perspectives from Interdisciplinary Teammates

**DOI:** 10.1093/jbcr/iraf019.516

**Published:** 2025-04-01

**Authors:** Thereasa Abrams, Aritra Moulick, Dana Dillard

**Affiliations:** University of Tennessee, Knoxville; University of Tennessee, Knoxville; Mississippi State University

## Abstract

**Introduction:**

There is little written about Social Work practice in domestic burn centers. Additionally, there are no standardized skills or guidelines required for Social Work burn care, resulting in considerable variability between burn centers. As the final phase in a three-part study investigating the roles, responsibilities, and impact of Social Work in burn care, the aim of this study was to better understand the demographics, resources, and perceptions of Social Work practice by multidisciplinary burn care teammates.

**Methods:**

The study employed a cross-sectional design to analyze descriptive statistics related to multidisciplinary burn care teammates’ perceptions of social work practice in burn centers. Structured surveys were distributed through ABA’s “listserv” that included institutional (number of ICU/step-down/pediatric beds, institutional ABA verification status, presence of social workers in respondents’ burn centers) and respondent (race, ethnicity, years working in burn care, gender, age) demographics and team members’ perception about social workers as members of multidisciplinary burn care teams.

Descriptive statistics were analyzed using SPSS. Frequency distributions and percentages were calculated for the categorical and binary variables such as race, ethnicity, geographic region, ABA verification status, population served, gender identity, presence and perceptions on social workers. Mean, median, mode, standard deviation, minimum, and maximum values were calculated for continuous variables such as number of ICU/step-down/pediatric beds and mean years worked in a burn center.

**Results:**

Survey results revealed that 53.7% of respondents (N=190) identified as female and 20.5% as male. More than half (62.6%) of the respondents identified as Caucasian, 3.7% as Asian, 1.1% as African American and 7.4% as Hispanic or Latino/a. Most burn centers (77.9%) were verified by the ABA, with nearly half (49.5%) serving both adult and pediatric patients. A significant number of respondents (75.3%) indicated their burn center has a designated social worker and 76.3% consider social workers to be integral members of multidisciplinary burn care teams.

**Conclusions:**

With the understanding that social workers are accepted members of most burn care teams, there is sound justification to employ designated social workers who are formally trained and ABA certified specifically for practice with burn patients and survivors in the same manner as other members of burn care teams.

**Applicability of Research to Practice:**

By increasing knowledge and understanding of Social Work’s role and impact in the care of burn patients, their families, and other members of the burn care team, future studies will support designating prepared social workers in every burn center.

**Funding for the Study:**

N/A

